# Identifying Issues in Effective Motor Imagery Training Practice in Children With Developmental Coordination Disorder: A Scoping Review

**DOI:** 10.1155/oti/9997678

**Published:** 2025-05-14

**Authors:** Akira Nakashima, Takuya Higasionnna, Yuto Iwanaga, Ryohei Okamura, Kengo Fujiwara, Toshio Higashi, Ryoichiro Iwanaga

**Affiliations:** ^1^Japan Society for the Promotion of Science, Tokyo, Japan; ^2^Graduate School of Biomedical Sciences, Nagasaki University, Nagasaki, Japan; ^3^Department of Rehabilitation, Faculty of Health Sciences, Tokyo Kasei University, Saitama, Japan; ^4^Department of Advanced Occupational Therapy, Human Health Sciences, Graduate School of Medicine, Kyoto University, Kyoto, Japan

## Abstract

**Introduction:** Motor imagery training (MIT) has gained attention as an occupational therapy tool for children with developmental coordination disorder (DCD). Although MIT has shown some effectiveness in children with DCD, intervention methods for DCD can still be improved. Further, occupational therapists should update their knowledge of motor imagery (MI) and MIT for children with DCD and understand their application.

**Objective:** The objective of this study is to survey the existing evidence on MI and MIT in children with DCD and comprehensively identify what is known and the problems that have been identified.

**Methods:** We used the Preferred Reporting Items for Systematic reviews and Meta-Analyses extension for Scoping Review and American Occupational Therapy Association guidelines for data collection and quality assessment. We searched for articles that included the words “developmental coordination disorder” and “motor imagery, motor imagery training (mental practice).” We searched the PubMed, Scopus, Medline, and the Cochrane Library databases; the search end date was March 12, 2024.

**Findings:** In total, 30 articles, including seven clinical studies and 23 fundamental studies, were eligible for this review. We surveyed (1) methods for assessing MI ability or clarity in children with DCD, (2) what is known about MI in children with DCD, and (3) the methodology of MIT for children with DCD.

**Conclusion:** Children with DCD have reduced MI ability compared to that had by typically developing children. Thus, MIT is recommended as a means of reducing the behavioral consequences of internal modeling deficits in children with DCD. On the other hand, information on the methodology used in MIT for children with DCD is inadequate, and no clear intervention measures have been proposed. In the future, it is important to clarify the amount of load when performing MIT and clarify the combination with other programs through more advanced research methods.

## 1. Introduction

Developmental coordination disorder (DCD) is a motor coordination disorder that occurs despite the absence of a disease [1, 2]. DCD is a diagnostic name established in the third edition of the *Diagnostic and Statistical Manual of Mental Disorders*. In some countries, an ongoing debate and sometimes confusion about the different terms and definitions used in this field exist. The term DCD is sometimes used with various definitions, and the terms that are similar to DCD are not always clear. For example, the Dyspraxia Foundation (UK) uses the term “dyspraxia,” stipulating that this term incorporates DCD. However, the definition provided is broader than that for DCD, as it includes various nonmotor difficulties. International consensus does not recommend the use of the term “dyspraxia” [1]. In the International Statistical Classification of Diseases and Related Health Problems-11, it is also described as developmental motor coordination disorder. DCD is prevalent in 5%–8% of school-aged children [1–3]. It is generally reported to cause problems with balance, coordination, writing, and other motor skills [4, 5], as well as with the necessary activities of daily living [6, 7]. In addition, children with DCD often have psychiatric disorders [8, 9] and are more likely to become socially withdrawn [10]. Therefore, there is a strong need for early rehabilitation to improve function and adaptation.

Interventions for motor skills [11–15], sports training [16–19], and Cognitive Orientation to daily Occupational Performance (CO-OP) [20–22] have been widely used in the rehabilitation of children with DCD. Under such circumstances, motor imagery training (MIT) has recently gained attention as a promising rehabilitation method for children with DCD. Motor imagery (MI) is defined as mental simulation or mental rehearsal of movement without actual movement [23, 24], and MIT is the repeated performance of MI to improve performance. Several studies have already reported the effectiveness of MIT in children with DCD [25–28].

One of the reasons for the attention on MIT for children with DCD is the internal model deficiency [29]. The internal model is the basic concept of motor control and motor learning [30, 31], but children with DCD have problems developing and implementing predictive models of behavior and tend to rely on feedback of inadequate results. This implies that motor learning requires significant time, and transfer of learning is difficult. Therefore, studies have used several methods, including mental rotation [32, 33], visually guided pointing [34], and sword [35, 36] tasks to assess MI; moreover, action planning ability has previously been assessed using a bar gasping task [37]. From a neurophysiological perspective, a study employing functional brain imaging techniques has revealed functional deterioration of the frontal–parietal lobe network and the cerebellum [38]. In such situations, MIT activates brain regions similar to those activated during actual movement [39] and is a useful intervention to promote neuroplastic changes in children with DCD [26]. Against this background, MIT for children with DCD is expected to be an effective rehabilitation tool to alleviate the effects of internal model deficits on activities of daily living and to help them acquire new movements.

To ensure effective MIT, it is important to conduct MI evaluations of children more clearly [40]. This necessitates an evaluation to determine whether the MI ability of the child with DCD is sufficient for implementing MIT (MI ability evaluation) and whether the child can perform the MI of the task clearly (MI vividness evaluation). However, the optimal method for assessing MI ability and MI vividness in children with DCD has not been clarified, and no studies have summarized the details of the differences in MI ability between typically developing (TD) children and children with DCD. In addition, to conduct effective MIT for children with DCD, it is necessary to establish a specialized MIT methodology for them that is different from that for other diseases and considers their unique conditions.

Therefore, in this scoping review, we focus on the current status of MI research in children with DCD, systematically mapping basic studies that have assessed and validated MI ability in children with DCD and clinical studies that have conducted MIT interventions or MI assessments. We aim to comprehensively clarify what has been discussed so far about MI skills and MIT in children with DCD and identify related issues, with the purpose of identifying research topics for establishing a methodology for MIT in children with DCD in the future.

## 2. Methods

Our scoping review methodology was originally conceived by Arksey and O'Malley [41], developed in detail by Levac et al. [42], and implemented based on the “Preferred Reporting Items for Systematic Reviews and Meta-Analyses Extension for Scoping Review” as compiled by Tricco et al. [43]. We structured our protocol by applying a four-step process: identifying the research question, identifying the studies, selecting the studies, and extracting and analyzing the data.

### 2.1. Step 1: Identifying the Research Question

The purpose of this study was to comprehensively clarify what is known so far about MI and MIT in children with DCD and the problems that have been identified, distinguishing between basic and clinical studies that have examined MI in children with DCD and the effects of MI or MIT. Specifically, we finalized the following questions: (1) How is MI ability or MI vividness assessed in addition to motor ability in studies of children with DCD? (2) What is known about MI in children with DCD? and (3) What methods are used to perform MIT in children with DCD? The PCOs that were used to identify research questions in this scoping review are listed in Table 1.

### 2.2. Step 2: Identifying Relevant Studies

We searched for articles that included the words “developmental coordination disorder” and “motor imagery, motor imagery training (mental practice).” The databases used were PubMed, Scopus, Medline, and the Cochrane Library; the last search date was March 12, 2024. Free-text terms and Boolean operators (AND/OR) were applied to search for titles or abstracts. No filters or limits were used. The keywords were selected to include studies in which MI or MIT was performed in children with DCD. The search strategy used for each database is shown in Table 2. Duplicate articles were removed from each database after the articles were extracted.

### 2.3. Step 3: Study Selection

The criteria for selecting eligible articles included the following: English language articles, with study design mentioned, and studies in which MI or MIT was performed for children with DCD. Selection of eligible articles was performed by four authors using the literature screening software Rayyan (https://www.rayyan.ai/). For each article, the first author (AN) and another author (TH, YI, or RI) checked whether the article met the eligibility criteria. In case of disagreement, the four authors reviewed the manuscript until 100% agreement was reached. Thereafter, AN and KF identified the levels of evidence and study designs of eligible articles using the *American Journal of Occupational Therapy's* systematic review guidelines (https://research.aota.org/DocumentLibrary/AOTA_AJOT_systematic%2520reviews%2520instructions.pdf).

### 2.4. Step 4: Data Extraction and Analysis

The following information was extracted from the eligible articles: author, year of publication, study type, study design, number of participants, age of participants, presence or absence of motor ability assessment, presence or absence of MI ability assessment, presence or absence of MI vividness assessment, other assessments, MIT intervention time, MIT intervention frequency, MIT intervention duration, MI task, and MIT implementation method.

## 3. Results

**Figure 1:**
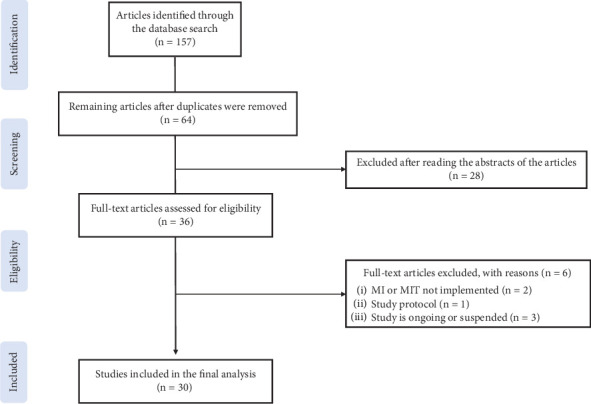
Flow diagram of study selection. *Note*. MI, motor imagery; MIT, motor imagery training. Figure format from “Preferred Reporting Items for Systematic Reviews and Meta-Analysis: The PRISMA Statement,” by D. Moher, A. Liberati, J. Tetzlaff, and D. G. Altman; PRISMA Group, 2009, PLoS Medicine, 6(7), e1000097. 10.1371/journal.pmed.1000097

In this study, 161 articles were identified, among which 30 were considered eligible (Figure 1). In all studies, the age of the children was 7–12 years. Of the 30 selected articles, seven were clinical studies, and 23 were basic studies (Table 3). One of the clinical studies examined the effects of a virtual reality (VR) intervention on children with DCD and employed MI ability in the evaluation. Two clinical studies were randomized controlled trials, and five were quasirandomized controlled trials.

### 3.1. How Is MI Ability or MI Vividness Assessed in Addition to Motor Ability in Studies of Children With DCD?

The results of all studies included showed the following: the Movement Assessment Battery for Children-Second Edition (MABC2) was used to assess motor skills in 14 studies, the Movement Assessment Battery for Children (MABC) in 12, the Developmental Coordination Disorder Questionnaire (DCDQ) in 10, the McCarron Assessment of Neuromuscular Development in 3, and the DCDQ 2007 in 1. Ten studies used more than one motor skill assessment. In the evaluation of MI ability, 14 studies used mental rotation tasks, eight studies used mental chronometry tasks, two studies used the Movement Imagery Questionnaire for Children (MIQ-C), and seven studies conducted no evaluation. The Florida Praxis Imagery Questionnaire (FPIQ) was used in only two fundamental studies to evaluate MI vividness. In the clinical studies, only four studies and all the studies did not assess MI ability and MI vividness, respectively. In the fundamental studies, MI ability was assessed in 20 articles, mostly via mental rotation or mental chronometry. Most of the mental rotations were conducted using hand images displayed on a PC screen, and the degree of MI ability was expressed using reaction time (RT) and percentage of correct responses as indices. Mental chronometry was assessed using a visual tracking task.

### 3.2. What Is Known About MI in Children With DCD?

Overall, 23 fundamental studies were analyzed for this purpose. Mental rotation or mental chronometry has been used to assess MI ability, and many studies have shown that MI ability is decreased in children with DCD compared to TD children. Lust et al. [51] reported no difference in MI ability between children with DCD and TD children as measured by RT in a hand mental rotation task, and others have reported that not all children with DCD show reduced MI ability [57] and that MI is affected only under conditions with complex task constraints [37].

To examine the effect of DCD severity on MI ability, children with DCD were divided into two groups, DCD mild (DCD-M) and DCD severe (DCD-S), and compared to TD children using MABC as an index. The results showed that the DCD-S group had a general decline in MI ability and did not show any improvement when given specific MI instructions, whereas the DCD-M group could perform simple MI and improved their MI ability due to specific instructions [52]. Regarding the development of MI ability, MI ability in children with DCD is lower, and the rate of development over time is comparable to that in TD children [58]. Another 2-year follow-up study reported that children with DCD developed MI more slowly and with less accuracy than that observed in TD children, but their MI ability improved over time and their action planning ability caught up with that of TD children [35]. Regarding the effects of the coexistence of DCD and other disorders, children with DCD and attention-deficit hyperactivity disorder (ADHD) showed a clear decline in MI, and their performance was as poor as that of children with DCD alone [33]. On the other hand, a study reported that MI ability was decreased in only children with DCD and that children with ADHD and DCD showed normal performance, albeit at a slower rate, in MI tasks, while no decrease in MI ability was observed in TD children or children with ADHD alone [53].

Many studies did not assess MI vividness. In a study of children with DCD and TD children, Fuchs and Caçola [59] reported that the FPIQ showed no difference in MI vividness between children with and without DCD.

Regarding how MI is performed, two studies examined the effect of combining MI with action observation (AO) and reported that combining MI with AO is more effective than using MI alone [61, 63].

### 3.3. What Methods Are Used to Perform MIT in Children With DCD?

Of the seven clinical studies, one included MI measures for evaluation. Therefore, the six studies in which the MIT intervention was implemented were analyzed. Of the six studies, two were randomized controlled trials, and four were quasirandomized controlled trials. Motor skills were evaluated using MABC, MABC2, and DCDQ, while the MIQ-C and mental chronometry were used in one study each to evaluate MI skills. Vividness of MI was not evaluated in all the studies. The MI tasks in MIT included motion tasks such as “catching a tennis ball” and “jumping” (three studies), activities of daily living such as “tying shoe laces” and “buttoning a shirt” (one study), and a visual tracking task (one study); task description was not provided in one study. The duration of MIT intervention was 5 min (one study), 10 min (one study), 40 min (one study), and 60 min (two studies). One study examined acute training effects within a single testing session that presented the number of sessions as 50. The weekly frequency of intervention was once a week in three studies and four times a week in two studies. The duration of intervention was 4 weeks in one study, 5 weeks in two studies, and 9 weeks in two studies. Regarding the method of MI recall during MIT, three studies combined MI with AO, and one study performed AO after MI. In this study, instructions for MI for each session were provided on a CD-ROM. In another study, MI was performed after AO in the first- and third-person viewpoints, and no guidance was provided during MI. Regarding the effects of MIT in children with DCD, MIT was described as effective in improving the motor skills of children with DCD [25, 28, 46], and two studies reported its usefulness as a home-based program involving family members [26, 27]. On the other hand, no protocols for intervention methods were provided [45].

## 4. Discussion

The purpose of this study was to comprehensively clarify what has been known so far about MI and MIT in children with DCD and what problems have been identified, distinguishing between basic studies and clinical studies that have examined MI in children with DCD and the effects of MI or MIT.

### 4.1. How Is MI Ability or MI Vividness Assessed in Addition to Motor Ability in Studies of Children With DCD?

Most MI ability assessments for children with DCD were conducted using mental rotation or mental chronometry, and only one study used the MIQ-C, a questionnaire-based assessment [26]. This may be because mental rotation, which can be implemented with easy explanations, is widely used in children because it is difficult to understand the assessment based on a questionnaire. However, compared with questionnaire evaluation, mental rotation is more susceptible to learning effects.

When conducting MI studies or interventions in sports or poststroke participants, MI ability is often assessed using questionnaires such as Kinesthetic and Visual Imagery Questionnaire-20 (KVIQ-20) [64] or Movement Imagery Questionnaire-Revised (MIQ-R) [65]. These evaluations are characterized by their ability to provide a detailed understanding of the participant's MI ability by dividing the evaluation into “visual MI” and “kinesthetic MI.” The KVIQ-20 is a 5-point scale for 10 different motor tasks, and the MIQ-R is a 7-point scale for seven different motor tasks. The higher the score for each evaluation, the higher the MI ability.

Only two studies have evaluated MI vividness using FPIQ. The MI vividness assessment in children with DCD did not question the vividness of the MI task. In sports and other diseases such as stroke, the vividness of MI is often measured using the visual analog scale (VAS) for MI tasks. The VAS is a method of evaluation in which the participant is asked to draw a line on a piece of paper representing the degree of clarity of the MI of an MI task (0 mm = not able to perform MI vividness at all for the task; 100 mm = able to perform enough MI vividness for the task). To implement an effective MIT, it is better to ask the MI vividness question for the target MI task.

The reasons for evaluating MI ability and vividness are (1) to screen whether an individual is eligible for MIT based on that individual's MI ability and (2) to confirm how well MI quality is ensured during MIT [66]. When implementing MIT for adults, the KVIQ-20 or MIQ-R is mostly used to assess MI ability before implementing MIT, but in children, there is currently no appropriate assessment. When considering methods for assessing MI ability in children in the future, an assessment using mental chronometry and mental rotation is affected by task-specific issues and learning, and it is appropriate to consider assessing MI ability in children using a questionnaire (e.g., MIQ-C), which is similar to that in adults. MIQ-C is an evaluation method based on the Movement Imagery Questionnaire-3 that has been improved for children [67], and it is characterized by its ability to evaluate MI ability by dividing it into internal visual imagery, external visual imagery, and kinesthetic imagery. It is expected to be widely used in the future implementation of MIT for children with DCD. In addition, it is important to clarify the MI task during MIT when evaluating MI vividness, and it is necessary to consider VAS and Likert scales for assessment.

### 4.2. What Is Known About MI in Children With DCD?

Many studies have revealed that children with DCD have reduced MI ability compared to that had by TD children. Research on TD children has shown that MI can be performed for children, beginning from age 6 or 7 years, and those children aged 10 years and above have the same MI ability as adults [68]. Kosslyn [69] explained that visual imagery consists of four processes, namely, generation, inspection, maintenance, and transformation, and suggests that some image transformations involve specific types of interactions between the motor and visual systems. Regarding the developmental period of each process, a study reported that the ability to generate images develops at age 8–9 years, the ability to maintain images develops at age 10–11 years, and the ability to maintain and transform images is integrated in adulthood [70]. Individual differences in MI ability are influenced by motor and cognitive abilities, specifically, executive function, planning ability, motor experience, working memory, and intelligence [71]. Regarding the difference in MI ability between children with DCD and TD children, the quality of MI ability was poorer in children with DCD than in TD children. However, similar to TD children, those with DCD improved their MI ability as they grew up [35, 58]. To date, no studies have analyzed and classified the MI abilities of children with DCD into generation, maintenance, and transformation; therefore, further investigation is needed.

On the other hand, some studies have reported that the MI ability of children with DCD is impaired only during complex tasks, suggesting that the details of the impairment are not yet fully understood. In addition, most of the studies on MI in children with DCD conducted to date have focused on mental chronometry and mental rotation, and very few studies have examined how poor MI affects the performance of children with DCD in physical activities such as jumping and activities of daily living. Until now, MIT research has focused solely on the impact of MIT on improving performance in athletes and individuals with upper limb paralysis after a stroke [62, 72]. In the future, it will be necessary to conduct research on how poor MI ability affects performance improvement in children with DCD from different perspectives, including from a basic perspective. Only two studies have investigated the neurophysiological relationship between MI ability and neurophysiology in children with DCD using an electroencephalogram and functional magnetic resonance imaging (MRI) [51, 73]. Till 2020, only 20 neuroimaging studies had been conducted using MRI in children with DCD [74], which may be because the participants are children and obtaining informed consent is difficult. However, it is important to conduct research on the role of the mirror neuron system [75–78] and visuomotor cognition [26, 79] in the learning abilities of children with DCD from a neurophysiological perspective. Future research should continue exploring these roles from various perspectives.

### 4.3. What Methods Are Used to Perform MIT in Children With DCD?

Many of the MITs for children with DCD were combined with AO when performing MI. In other areas, such as sports, combining AO with MI has been reported to increase effectiveness [80]. Especially for children with reduced MI ability compared to that of adults, we speculate that a combination of AO may be more effective [46, 71]. Unlike MIT for sports and patients with stroke, MIT for children with DCD does not achieve or insufficiently acquire the task movements of MI [26]. Therefore, in addition to the problem that children with DCD cannot receive sufficient internal feedback in movement execution [6], they may also be unable to obtain sufficient knowledge of the results (KRs) [81] and knowledge of performance (KP) [82] of the target movement as external feedback. Feedback is the sensory information needed to acquire motor skills and is most effective when it provides information about the correct response, not the incorrect response [83]. Although both internal and external focus are effective methods of directing motor learning for children with DCD, these methods are reportedly more effective in children with high visuospatial working memory when their attention is directed to external focus [84]. The advantage of combining AO with MI has been used to improve the vividness of MI, but for children with DCD, it may have the effect of ensuring that internal feedback and feedback by KR and KP can be obtained more reliably, in addition to ensuring MI vividness. Since the frequency of feedback influences motor learning [85], it may be necessary to consider a feedback method that differs from the usual MIT and is appropriate for children with DCD. Based on this background, we believe it is necessary to conduct further research on issues such as the combination of AO during MIT for children with DCD, if AO should be performed during MI or before MI, whether first-person AO is better, or whether the third-person viewpoint is better to ensure the visualization of the entire body. Study methodologies should also be evaluated for such issues. In addition, several studies have recently begun to use VR and brain–computer interface to improve MI vividness, and it will be important to apply MI vividness techniques to optimize the effectiveness of MIT in the future. One of the therapies that is becoming more and more effective with VR technology is mirror therapy (MT). MT induces cortical reorganization, promotes plastic changes in the brain without moving the affected limb, and has already been proven effective [86, 87]. In this context, the VR-based MT system (VRMT), which applies the MT concept, is expected to be an effective treatment method compared to conventional MT [88, 89]. Systematic reviews have already reported that VRMT is effective when combined with conventional rehabilitation [90]. We believe that the application of this technology can be fully utilized in MIT for children with DCD.

There were no fixed standards for the amount of MIT, which varied from study to study. A similar trend has been observed in people with stroke, and a standard for the MIT loading dose is needed [91–93]. In addition, it has recently been shown that fatigue appears with sustained repetition of MI, which has been reported to affect performance [94–97]. With this background, paying sufficient attention to the amount of load when MIT is performed, especially in children, is necessary. In the future, it will be necessary to consider the time, frequency, and duration of MIT interventions appropriate for children from the viewpoint of performance improvement.

Rehabilitation of children with DCD can be divided into process- or deficit-oriented and task-specific rehabilitation [98]. In general, process- or deficit-oriented interventions focus on improving physical function, while task-specific interventions focus on the specific motor task that is problematic for the child. Specifically, process- or deficit-oriented interventions refer to sensory integration therapies [99], and task-specific interventions refer to task-oriented approaches [100, 101], neuromotor task training [102, 103], and CO-OP [104, 105]. In MIT for children with DCD, some studies used an MI task that was a physical activity such as jumping, while some studies used an MI task that was an activity of daily living such as buttoning or tying shoelaces. It is unclear from the studies whether MIT was deficit-oriented or task-specific, and it is necessary to examine what kind of performance changes can be derived in the future. Regarding the MIT approach, MIT for upper limb function on the paralyzed side of patients with stroke was combined with exercise therapy in all studies, and MIT is considered a complementary intervention to exercise therapy. In fact, combining MIT with exercise therapy has been reported to help improve performance [106, 107]. On the other hand, many studies have used only MIT for children with DCD due to disease-specific or environmental influences. Scott et al. provided their target children with real-life training of the task behavior after the implementation of MIT [26]. In the future, it will be important to examine effective MIT implementation methods by combining actual operation training into a single package and to verify the effects of time allocation between MIT and actual operation training and verify the timing of actual operation training.

## 5. Limitations

As this was a scoping review, we did not evaluate the advantages and disadvantages of MIT in each study. Thus, it was not possible to describe the effectiveness of MIT in occupational therapy interventions. In addition, although four experienced occupational therapists reviewed each study, we cannot deny the possibility that other occupational therapists or a different team of occupational therapists would have had a different opinion. Furthermore, many of the studies included in this study were of low quality; therefore, the results of this study should be interpreted with caution.

## 6. Future Research Directions

This scoping review revealed what is known about MI in children with DCD and the effect of MIT on improving motor skills and activities of daily living in children with DCD while identifying items that need to be considered in future research. First, we need to establish evidence using evaluation methods such as MI ability and MI vividness for children with DCD and the neurophysiological methods for MI. Second, we need to understand how to combine AO to enhance the effectiveness of MIT. Third, we need to establish a methodology for combining MIT with exercise therapy and other therapies. Although MIT for children with DCD differs in many respects from the MIT that has been conducted in the fields of sports, cerebrovascular disease, and orthopedic disease, the effectiveness of MIT for children with DCD is expected to be sufficient, and we believe that the development of research in this field will sufficiently contribute to occupational therapy for children with DCD.

## 7. Conclusion

This scoping review identified several issues that are necessary for more effective MIT practices in children with DCD to provide a guidepost for future research. In the future, we believe that basic and clinical researchers should cooperate with each other to accumulate data, which will lead to solving the problems identified in this scoping review and contribute to establishing a methodology for MIT specific to children with DCD.

## Figures and Tables

**Table 1:** Identifying the research question and inclusion criteria.

**Identifying the research question**	
Participants	DCD in children
Concept	Motor imagery, motor imagery training, evaluation items, method, intervention periods
Context	Abroad

**Inclusion criteria**	
• A study of MI or MIT for DCD in children (adopt all study types) • English papers	

Abbreviations: DCD, developmental coordination disorder; MI, motor imagery; MIT, motor imagery training.

**Table 2:** Full search strategies for each database.

**Database**	**Search strategy**
PubMed	(“mental practice”[Title/Abstract] OR “motor imagery training”[Title/Abstract] OR “motor imagery”[Title/Abstract] OR “motor image”[Title/Abstract]) AND (“developmental coordination disorder”[Title/Abstract] OR “apraxia”[Title/Abstract] OR “DCD”[Title/Abstract] OR “clumsy”[Title/Abstract] OR “Nonverbal Learning Disorder”[Title/Abstract] OR “Awkward”[Title/Abstract] OR “motor skills disorder”[Title/Abstract] OR “dyspraxia”[Title/Abstract]”) AND (“children”[Title/Abstract] OR “infants”[Title/Abstract] OR “toddlers”[Title/Abstract] OR “school children”[Title/Abstract])
Scopus	(TITLE-ABS-KEY (“mental practice” OR “motor imagery training” OR “motor imagery” OR “motor image”) AND TITLE-ABS-KEY (“developmental coordination disorder” OR “apraxia” OR “dcd” OR “clumsy” OR “nonverbal learning disorder” OR “awkward” OR “motor skills disorder” OR “dyspraxia”) AND TITLE-ABS-KEY (“children” OR “infants” OR “toddlers” OR “school children”))
Medline	Tiab(“mental practice” OR “motor imagery training” OR “motor imagery” OR “motor image”) AND tiab(“developmental coordination disorder” OR “apraxia” OR “DCD” OR “clumsy” OR “Nonverbal Learning Disorder” OR “Awkward” OR “motor skills disorder” OR “dyspraxia”) AND tiab(“children” OR “infants” OR “toddlers” OR “school children”)
Cochrane Library	Cochrane Reviews matching “mental practice” OR “motor imagery training” OR “motor imagery” OR “motor image” in Title Abstract Keyword AND “developmental coordination disorder” OR “apraxia” OR “DCD” OR “clumsy” OR “Nonverbal Learning Disorder” OR “Awkward” OR “motor skills disorder” OR “dyspraxia” in Title Abstract Keyword AND “children” OR “infants” OR “toddlers” OR “school children” in Title Abstract Keyword

**Table 3:** Levels of evidence and evaluation of motor imagery ability and vividness for the articles included in this scoping review.

**No.**	**Citation**	**Evidence level and study design**	**Evaluation of MI ability**	**Evaluation of MI vividness**
Clinical research				
1	Wilson et al. [25]	2B/low-quality RCT	NA	NA
2	Wilson et al. [28]	2B/low-quality RCT	NA	NA
3	Adams et al. [27]	2B/two-group, nonrandomized	NA	NA
4	EbrahimiSani et al. [44]	2B/two-group, nonrandomized	MR	NA
5	Ganapathy et al. [45]	2B/two-group, nonrandomized	NA	NA
6	Marshall et al. [46]	2B/two-group, nonrandomized	MC	NA
7	Scott et al. [26]	2B/two-group, nonrandomized	MIQ-C	NA
Fundamental research				
8	Wilson et al. [47]	NA	MC	FPIQ
9	Katschmarsky et al. [48]	NA	MC	NA
10	Wilson et al. [49]	NA	MR	NA
11	Williams et al. [50]	NA	MR	NA
12	Lust et al. [51]	NA	MR	NA
13	Williams et al. [52]	NA	MR	NA
14	Lewis et al. [53]	NA	MC	NA
15	Deconinck et al. [32]	NA	MR	NA
16	Williams et al. [54]	NA	MR	NA
17	Williams et al. [33]	NA	MR &MC	NA
18	Cacola et al. [55]	NA	MC	NA
19	Noten et al. [37]	NA	MR	NA
20	Fuelscher et al. [56]	NA	MR	NA
21	Reynolds et al. [57]	NA	MR	NA
22	Ferguson et al. [34]	NA	MC	NA
23	Adams et al. [35]	NA	MR	NA
24	Adams et al. [36]	NA	MR	NA
25	Adams et al. [58]	NA	MC	NA
26	Fuchs and Caçola [59]	NA	MIQ-C	FPIQ
27	Bhoyroo et al. [60]	NA	NA	NA
28	Scott et al. [61]	NA	NA	NA
29	Page and Levine et al. [62]	NA	MR	NA
30	Scott et al. [63]	NA	NA	NA

Abbreviations: FPIQ, Florida Praxis Imagery Questionnaire; MC, mental chronometry; MI, motor imagery; MIQ-C, Movement Imagery Questionnaire for Children; MR, mental rotation; NA, not available; RCT, randomized controlled trial.

## Data Availability

Research data are not shared.

## References

[1] Blank R., Barnett A. L., Cairney J. (2019). International Clinical Practice Recommendations on the Definition, Diagnosis, Assessment, Intervention, and Psychosocial Aspects of Developmental Coordination Disorder. *Developmental Medicine and Child Neurology*.

[2] American Psychiatric Association (2020). *Diagnostic and Statistical Manual of Mental Disorders: Text Revision (DSM-5-TR).*.

[3] World Health Organization (2010). *International Statistical Classification of Diseases and Related Health Problems*.

[4] Nakai A., Miyachi T., Okada R. (2011). Evaluation of the Japanese Version of the Developmental Coordination Disorder Questionnaire as a Screening Tool for Clumsiness of Japanese Children. *Research in Developmental Disabilities*.

[5] Vaivre-Douret L., Lalanne C., Ingster-Moati I. (2011). Subtypes of Developmental Coordination Disorder: Research on Their Nature and Etiology. *Developmental Neuropsychology*.

[6] Wilson P. H., Ruddock S., Smits-Engelsman B., Polatajko H., Blank R. (2013). Understanding Performance Deficits in Developmental Coordination Disorder: A Meta-Analysis of Recent Research. *Developmental Medicine and Child Neurology*.

[7] Adams I. L. J., Ferguson G. D., Lust J. M., Steenbergen B., Smits-Engelsman B. C. M. (2016). Action Planning and Position Sense in Children With Developmental Coordination Disorder. *Human Movement Science*.

[8] Cermak S. A., Katz N., Weintraub N. (2015). Participation in Physical Activity, Fitness, and Risk for Obesity in Children With Developmental Coordination Disorder: A Cross-Cultural Study. *Occupational Therapy International*.

[9] Lingam R., Jongmans M. J., Ellis M., Hunt L. P., Golding J., Emond A. (2012). Mental Health Difficulties in Children With Developmental Coordination Disorder. *Pediatrics*.

[10] Sylvestre A., Nadeau L., Charron L., Larose N., Lepage C. (2013). Social Participation by Children With Developmental Coordination Disorder Compared to Their Peers. *Disability and Rehabilitation*.

[11] Au M. K., Chan W. M., Lee L., Chen T. M. K., Chau R. M. W., Pang M. Y. C. (2014). Core Stability Exercise is as Effective as Task-Oriented Motor Training in Improving Motor Proficiency in Children With Developmental Coordination Disorder: A Randomized Controlled Pilot Study. *Clinical Rehabilitation*.

[12] Fong S. S. M., Guo X., Liu K. P. Y. (2016). Task-Specific Balance Training Improves the Sensory Organisation of Balance Control in Children With Developmental Coordination Disorder: A Randomised Controlled Trial. *Scientific Reports*.

[13] Dannenbaum E., Bégin C. L., Daigneault-Bourgeois É. (2022). Feasibility and Preliminary Effects of a 1-Week Vestibular Rehabilitation Day Camp in Children With Developmental Coordination Disorder. *Physical & Occupational Therapy in Pediatrics*.

[14] Andelin L., Reynolds S., Schoen S. (2021). Effectiveness of Occupational Therapy Using a Sensory Integration Approach: A Multiple-Baseline Design Study. *American Journal of Occupational Therapy*.

[15] Fong S. S. M., Chung L. M. Y., Schooling C. M. (2022). Tai Chi-Muscle Power Training for Children With Developmental Coordination Disorder: A Randomized Controlled Trial. *Scientific Reports*.

[16] Fong S. S. M., Chung J. W. Y., Chow L. P. Y., Ma A. W. W., Tsang W. W. N. (2013). Differential Effect of Taekwondo Training on Knee Muscle Strength and Reactive and Static Balance Control in Children With Developmental Coordination Disorder: A Randomized Controlled Trial. *Research in Developmental Disabilities*.

[17] Hillier S., McIntyre A., Plummer L. (2010). Aquatic Physical Therapy for Children With Developmental Coordination Disorder: A Pilot Randomized Controlled Trial. *Physical and Occupational Therapy in Pediatrics*.

[18] Ma A. W. W., Fong S. S. M., Guo X. (2018). Adapted Taekwondo Training for Prepubertal Children With Developmental Coordination Disorder: A Randomized, Controlled Trial. *Scientific Reports*.

[19] Cavalcante Neto J. L., Steenbergen B., Tudella E. (2019). Motor Intervention With and Without Nintendo Wii for Children With Developmental Coordination Disorder: Protocol for a Randomized Clinical Trial. *Trials*.

[20] Araujo C. R. S., Cardoso A. A., Polatajko H. J., de Castro Magalhães L. (2021). Efficacy of the Cognitive Orientation to Daily Occupational Performance (CO-OP) Approach With and Without Parental Coaching on Activity and Participation for Children With Developmental Coordination Disorder: A Randomized Clinical Trial. *Research in Developmental Disabilities*.

[21] Krajenbrink H., Lust J., van Heeswijk J., Aarts P., Steenbergen B. (2022). Benefits of an Intensive Individual CO-OP Intervention in a Group Setting for Children With DCD. *Occupational Therapy International*.

[22] Thornton A., Licari M., Reid S., Armstrong J., Fallows R., Elliott C. (2016). Cognitive Orientation to (Daily) Occupational Performance Intervention Leads to Improvements in Impairments, Activity and Participation in Children With Developmental Coordination Disorder. *Disability and Rehabilitation*.

[23] Jeannerod M. (1995). Mental Imagery in the Motor Context. *Neuropsychologia*.

[24] Decety J. (1996). The Neurophysiological Basis of Motor Imagery. *Behavioural Brain Research*.

[25] Wilson P. H., Thomas P. R., Maruff P. (2002). Motor Imagery Training Ameliorates Motor Clumsiness in Children. *Journal of Child Neurology*.

[26] Scott M. W., Wood G., Holmes P. S., Marshall B., Williams J., Wright D. J. (2023). Combined Action Observation and Motor Imagery Improves Learning of Activities of Daily Living in Children With Developmental Coordination Disorder. *PLoS One*.

[27] Adams I. L. J., Smits-Engelsman B., Lust J. M., Wilson P. H., Steenbergen B. (2017). Feasibility of Motor Imagery Training for Children With Developmental Coordination Disorder – A Pilot Study. *Frontiers in Psychology*.

[28] Wilson P. H., Adams I. L. J., Caeyenberghs K., Thomas P., Smits-Engelsman B., Steenbergen B. (2016). Motor Imagery Training Enhances Motor Skill in Children With DCD: A Replication Study. *Research in Developmental Disabilities*.

[29] Adams I. L. J., Lust J. M., Wilson P. H., Steenbergen B. (2014). Compromised Motor Control in Children With DCD: A Deficit in the Internal Model?—A Systematic Review. *Neuroscience and Biobehavioral Reviews*.

[30] Wolpert D. M. (1997). Computational Approaches to Motor Control. *Trends in Cognitive Sciences*.

[31] Jeannerod M. (2001). Neural Simulation of Action: A Unifying Mechanism for Motor Cognition. *NeuroImage*.

[32] Deconinck F. J. A., Spitaels L., Fias W., Lenoir M. (2009). Is Developmental Coordination Disorder a Motor Imagery Deficit?. *Journal of Clinical and Experimental Neuropsychology*.

[33] Williams J., Omizzolo C., Galea M. P., Vance A. (2013). Motor Imagery Skills of Children With Attention Deficit Hyperactivity Disorder and Developmental Coordination Disorder. *Human Movement Science*.

[34] Ferguson G. D., Wilson P. H., Smits-Engelsman B. C. M. (2015). The Influence of Task Paradigm on Motor Imagery Ability in Children With Developmental Coordination Disorder. *Human Movement Science*.

[35] Adams I. L. J., Lust J. M., Wilson P. H., Steenbergen B. (2017). Development of Motor Imagery and Anticipatory Action Planning in Children With Developmental Coordination Disorder - A Longitudinal Approach. *Human Movement Science*.

[36] Adams I. L. J., Lust J. M., Wilson P. H., Steenbergen B. (2017). Testing Predictive Control of Movement in Children With Developmental Coordination Disorder Using Converging Operations. *British Journal of Psychology*.

[37] Noten M., Wilson P., Ruddock S., Steenbergen B. (2014). Mild Impairments of Motor Imagery Skills in Children With DCD. *Research in Developmental Disabilities*.

[38] Zwicker J. G., Missiuna C., Harris S. R., Boyd L. A. (2010). Brain Activation of Children With Developmental Coordination Disorder is Different Than Peers. *Pediatrics*.

[39] Hétu S., Grégoire M., Saimpont A. (2013). The Neural Network of Motor Imagery: An ALE Meta-Analysis. *Neuroscience & Biobehavioral Reviews*.

[40] Ruffino C., Papaxanthis C., Lebon F. (2017). The Influence of Imagery Capacity in Motor Performance Improvement. *Experimental Brain Research*.

[41] Arksey H., O'Malley L. (2005). Scoping Studies: Towards a Methodological Framework. *International Journal of Social Research Methodology*.

[42] Levac D., Colquhoun H., O'Brien K. K. (2010). Scoping studies: Advancing the Methodology. *Implementation Science*.

[43] Tricco A. C., Lillie E., Zarin W. (2018). PRISMA Extension for Scoping Reviews (PRISMA-ScR): Checklist and Explanation. *Annals of Internal Medicine*.

[44] EbrahimiSani S., Sohrabi M., Taheri H., Agdasi M. T., Amiri S. (2020). Effects of Virtual Reality Training Intervention on Predictive Motor Control of Children With DCD – A Randomized Controlled Trial. *Research in Developmental Disabilities*.

[45] Ganapathy Sankar U., Monisha R. (2020). Effectiveness of Motor Imagery Training for Children With Developmental Coordination Disorder Among Indian Children- A Pilot Study. *International Journal of Research in Pharmacy Science*.

[46] Marshall B., Wright D. J., Holmes P. S., Williams J., Wood G. (2020). Combined Action Observation and Motor Imagery Facilitates Visuomotor Adaptation in Children With Developmental Coordination Disorder. *Research in Developmental Disabilities*.

[47] Wilson P. H., Maruff P., Ives S., Currie J. (2001). Abnormalities of Motor and Praxis Imagery in Children With DCD. *Human Movement Science*.

[48] Katschmarsky S., Cairney S., Maruff P., Wilson P. H., Currie J. (2001). The Ability to Execute Saccades on the Basis of Efference Copy: Impairments in Double-Step Saccade Performance in Children With Developmental Co-Ordination Disorder. *Experimental Brain Research*.

[49] Wilson P. H., Maruff P., Butson M., Williams J., Lum J., Thomas P. R. (2004). Internal Representation of Movement in Children With Developmental Coordination Disorder: A Mental Rotation Task. *Developmental Medicine and Child Neurology*.

[50] Williams J., Thomas P. R., Maruff P., Butson M., Wilson P. H. (2006). Motor, Visual and Egocentric Transformations in Children With Developmental Coordination Disorder. *Child: Care, Health and Development*.

[51] Lust J. M., Geuze R. H., Wijers A. A., Wilson P. H. (2006). An EEG Study of Mental Rotation-Related Negativity in Children With Developmental Coordination Disorder. *Child: Care, Health & Development*.

[52] Williams J., Thomas P. R., Maruff P., Wilson P. H. (2008). The Link Between Motor Impairment Level and Motor Imagery Ability in Children With Developmental Coordination Disorder. *Human Movement Science*.

[53] Lewis M., Vance A., Maruff P., Wilson P., Cairney S. (2008). Differences in Motor Imagery Between Children With Developmental Coordination Disorder With and Without the Combined Type of ADHD. *Developmental Medicine and Child Neurology*.

[54] Williams J., Anderson V., Reddihough D. S., Reid S. M., Vijayakumar N., Wilson P. H. (2011). A Comparison of Motor Imagery Performance in Children With Spastic Hemiplegia and Developmental Coordination Disorder. *Journal of Clinical and Experimental Neuropsychology*.

[55] Caçola P., Gabbard C., Ibana M., Romero M. (2014). Tool Length Influences Reach Distance Estimation via Motor Imagery in Children With Developmental Coordination Disorder. *Journal of Clinical and Experimental Neuropsychology*.

[56] Fuelscher I., Williams J., Enticott P. G., Hyde C. (2015). Reduced Motor Imagery Efficiency Is Associated With Online Control Difficulties in Children With Probable Developmental Coordination Disorder. *Research in Developmental Disabilities*.

[57] Reynolds J. E., Licari M. K., Elliott C., Lay B. S., Williams J. (2015). Motor Imagery Ability and Internal Representation of Movement in Children With Probable Developmental Coordination Disorder. *Human Movement Science*.

[58] Adams I. L. J., Lust J. M., Steenbergen B. (2018). Development of Motor Imagery Ability in Children With Developmental Coordination Disorder - A Goal-Directed Pointing Task. *British Journal of Psychology*.

[59] Fuchs C. T., Caçola P. (2018). Differences in Accuracy and Vividness of Motor Imagery in Children With and Without Developmental Coordination Disorder. *Human Movement Science*.

[60] Bhoyroo R., Hands B., Wilmut K., Hyde C., Wigley A. (2019). Motor Planning With and Without Motor Imagery in Children With Developmental Coordination Disorder. *Acta Psychologica*.

[61] Scott M. W., Emerson J. R., Dixon J., Tayler M. A., Eaves D. L. (2019). Motor Imagery During Action Observation Enhances Automatic Imitation in Children With and Without Developmental Coordination Disorder. *Journal of Experimental Child Psychology*.

[62] Page S. J., Levine P. (2021). Multimodal Mental Practice Versus Repetitive Task Practice Only to Treat Chronic Stroke: A Randomized Controlled Pilot Study. *American Journal of Occupational Therapy*.

[63] Scott M. W., Emerson J. R., Dixon J., Tayler M. A., Eaves D. L. (2020). Motor Imagery During Action Observation Enhances Imitation of Everyday Rhythmical Actions in Children With and Without Developmental Coordination Disorder. *Human Movement Science*.

[64] Malouin F., Richards C. L., Jackson P. L., Lafleur M. F., Durand A., Doyon J. (2007). The Kinesthetic and Visual Imagery Questionnaire (KVIQ) for Assessing Motor Imagery in Persons With Physical Disabilities: A reliability and Construct Validity Study. *Journal of Neurologic Physical Therapy*.

[65] Hall C. R., Martin K. A. (1997). Measuring Movement Imagery Abilities: A Revision of the Movement Imagery Questionnaire. *Journal of Mental Imagery*.

[66] Di Rienzo F., Collet C., Hoyek N., Guillot A. (2014). Impact of Neurologic Deficits on Motor Imagery: A Systematic Review of Clinical Evaluations. *Neuropsychology Review*.

[67] Williams S. E., Cumming J., Ntoumanis N., Nordin-Bates S. M., Ramsey R., Hall C. (2012). Further Validation and Development of the Movement Imagery Questionnaire. *Journal of Sport and Exercise Psychology*.

[68] Souto D. O., Cruz T. K. F., Fontes P. L. B., Batista R. C., Haase V. G. (2020). Motor Imagery Development in Children: Changes in Speed and Accuracy With Increasing Age. *Frontiers in Pediatrics*.

[69] Kosslyn S. M. (1994). *Image and Brain: The Resolution of the Imagery Debate*.

[70] Bates K. E., Farran E. K. (2021). Mental Imagery and Visual Working Memory Abilities Appear to be Unrelated in Childhood: Evidence for Individual Differences in Strategy Use. *Cognitive Development*.

[71] Spruijt S., van der Kamp J., Steenbergen B. (2015). Current Insights in the Development of Children’s Motor Imagery Ability. *Frontiers in Psychology*.

[72] Mizuguchi N., Nakata H., Uchida Y., Kanosue K. (2012). Motor Imagery and Sport Performance. *Journal of Physical Fitness and Sports Medicine*.

[73] Reynolds J. E., Billington J., Kerrigan S. (2019). Mirror Neuron System Activation in Children With Developmental Coordination Disorder: A Replication Functional MRI Study. *Research in Developmental Disabilities*.

[74] Irie K., Matsumoto A., Zhao S., Kato T., Liang N. (2021). Neural Basis and Motor Imagery Intervention Methodology Based on Neuroimaging Studies in Children With Developmental Coordination Disorders: A Review. *Frontiers in Human Neuroscience*.

[75] Werner J. M., Cermak S. A., Aziz-Zadeh L. (2012). Neural Correlates of Developmental Coordination Disorder: The Mirror Neuron System Hypothesis. *Journal of Behavioral and Brain Science*.

[76] Licari M. K., Billington J., Reid S. L. (2015). Cortical Functioning in Children With Developmental Coordination Disorder: A Motor Overflow Study. *Experimental Brain Research*.

[77] Reynolds J. E., Licari M. K., Billington J. (2015). Mirror Neuron Activation in Children With Developmental Coordination Disorder: A Functional MRI Study. *International Journal of Developmental Neuroscience*.

[78] Reynolds J. E., Thornton A. L., Elliott C., Williams J., Lay B. S., Licari M. K. (2015). A Systematic Review of Mirror Neuron System Function in Developmental Coordination Disorder: Imitation, Motor Imagery, and Neuroimaging Evidence. *Research in Developmental Disabilities*.

[79] Scott M. W., Wood G., Holmes P. S., Williams J., Marshall B., Wright D. J. (2021). Combined Action Observation and Motor Imagery: An Intervention to Combat the Neural and Behavioural Deficits Associated With Developmental Coordination Disorder. *Neuroscience and Biobehavioral Reviews*.

[80] Eaves D. L., Riach M., Holmes P. S., Wright D. J. (2016). Motor Imagery During Action Observation: A Brief Review of Evidence, Theory and Future Research Opportunities. *Frontiers in Neuroscience*.

[81] Salmoni A. W., Schmidt R. A., Walter C. B. (1984). Knowledge of Results and Motor Learning: A Review and Critical Reappraisal. *Psychological Bulletin*.

[82] Brisson T. A., Alain C. (1997). A Comparison of Two References for Using Knowledge of Performance in Learning a Motor Task. *Journal of Motor Behavior*.

[83] Losada M., Heaphy E. (2004). The Role of Positivity and Connectivity in the Performance of Business Teams: A Nonlinear Dynamics Model. *American Behavioral Scientist*.

[84] van Cappellen-van Maldegem S. J. M., van Abswoude F., Krajenbrink H., Steenbergen B. (2018). Motor Learning in Children With Developmental Coordination Disorder: The Role of Focus of Attention and Working Memory. *Human Movement Science*.

[85] Sullivan K. J., Kantak S. S., Burtner P. A. (2008). Motor Learning in Children: Feedback Effects on Skill Acquisition. *Physical Therapy*.

[86] Arya K. N. (2016). Underlying Neural Mechanisms of Mirror Therapy: Implications for Motor Rehabilitation in Stroke. *Neurology India*.

[87] Thieme H., Morkisch N., Mehrholz J. (2018). Mirror Therapy for Improving Motor Function After Stroke. *Cochrane Database of Systematic Reviews*.

[88] Kim J., Yi J., Song C.-H. (2017). Kinematic Analysis of Head, Trunk, and Pelvic Motion During Mirror Therapy for Stroke Patients. *Journal of Physical Therapy Science*.

[89] Kim J., Song C. (2021). Postural Difference Between the Interventions Reflecting the Concept of Mirror Therapy in Healthy Subjects. *Brain Sciences*.

[90] Okamura R., Nakashima A., Moriuchi T. (2023). Effects of a Virtual Reality-Based Mirror Therapy System on Upper Extremity Rehabilitation After Stroke: A Systematic Review and Meta-Analysis of Randomized Controlled Trials. *Frontiers in Neurology*.

[91] Malouin F., Jackson P. L., Richards C. L. (2013). Towards the Integration of Mental Practice in Rehabilitation Programs. A Critical Review. *Frontiers in Human Neuroscience*.

[92] Guerra Z. F., Lucchetti A. L. G., Lucchetti G. (2017). Motor Imagery Training After Stroke: A Systematic Review and Meta-Analysis of Randomized Controlled Trials. *Journal of Neurologic Physical Therapy*.

[93] Nakashima A., Okamura R., Moriuchi T., Fujiwara K., Higashi T., Tomori K. (2024). Exploring Methodological Issues in Mental Practice for Upper-Extremity Function Following Stroke-Related Paralysis: A Scoping Review. *Brain Sciences*.

[94] Graham J. D., Sonne M. W. L., Bray S. R. (2014). It Wears Me Out Just Imagining It! Mental Imagery Leads to Muscle Fatigue and Diminished Performance of Isometric Exercise. *Biological Psychology*.

[95] Rozand V., Lebon F., Stapley P. J., Papaxanthis C., Lepers R. (2016). A Prolonged Motor Imagery Session Alter Imagined and Actual Movement Durations: Potential Implications for Neurorehabilitation. *Behavioural Brain Research*.

[96] Nakashima A., Moriuchi T., Matsuda D. (2021). Corticospinal Excitability During Motor Imagery is Diminished by Continuous Repetition-Induced Fatigue. *Neural Regeneration Research*.

[97] Nakashima A., Moriuchi T., Matsuda D. (2022). Continuous Repetition Motor Imagery Training and Physical Practice Training Exert the Growth of Fatigue and Its Effect on Performance. *Brain Sciences*.

[98] Zwicker J. G., Missiuna C., Harris S. R., Boyd L. A. (2012). Developmental Coordination Disorder: A Review and Update. *European Journal of Paediatric Neurology*.

[99] Section On Complementary And Integrative Medicine, Council on Children with Disabilities, American Academy of Pediatrics, Zimmer M., Desch L. (2012). Sensory Integration Therapies for Children With Developmental and Behavioral Disorders. *Pediatrics*.

[100] Jongmans M. J., Linthorst-Bakker E., Westenberg Y., Smits-Engelsman B. C. M. (2003). Use of a Task-Oriented Self-Instruction Method to Support Children in Primary School With Poor Handwriting Quality and Speed. *Human Movement Science*.

[101] Dunford C. (2011). Goal-Orientated Group Intervention for Children With Developmental Coordination Disorder. *Physical and Occupational Therapy in Pediatrics*.

[102] Schoemaker M. M., Niemeijer A. S., Reynders K., Smits-Engelsman B. C. M. (2003). Effectiveness of Neuromotor Task Training for Children With Developmental Coordination Disorder: A Pilot Study. *Neural Plasticity*.

[103] Niemeijer A. S., Smits-Engelsman B. C. M., Schoemaker M. M. (2007). Neuromotor Task Training for Children With Developmental Coordination Disorder: A Controlled Trial. *Developmental Medicine and Child Neurology*.

[104] Ward A., Rodger S. (2004). The Application of Cognitive Orientation to Daily Occupational Performance (CO-OP) With Children 5–7 Years With Developmental Coordination Disorder. *British Journal of Occupational Therapy*.

[105] Anderson L., Wilson J., Williams G. (2017). Cognitive Orientation to Daily Occupational Performance (CO-OP) as Group Therapy for Children Living With Motor Coordination Difficulties: An Integrated Literature Review. *Australian Occupational Therapy Journal*.

[106] Stockley R. C., Jarvis K., Boland P., Clegg A. J. (2021). Systematic Review and Meta-Analysis of the Effectiveness of Mental Practice for the Upper Limb After Stroke: Imagined or Real Benefit?. *Archives of Physical Medicine and Rehabilitation*.

[107] Villa-Berges E., Laborda Soriano A. A., Lucha-López O. (2023). Motor Imagery and Mental Practice in the Subacute and Chronic Phases in Upper Limb Rehabilitation After Stroke: A Systematic Review. *Occupational Therapy International*.

